# Supplementary material for: The RING-finger domain of Arabidopsis RMR functions as an E3 ligase essential for post-Golgi trafficking
Source: J Biol Chem. 2025 Sep 15;301(10):110721. doi: 10.1016/j.jbc.2025.110721 (PMC12552968; doi:10.1016/j.jbc.2025.110721)
Supplement: Supporting information [file mmc1.pdf]

Supporting Information for

The RING-finger domain of *Arabidopsis* RMR functions as an E3 ligase essential for post-Golgi trafficking

Shuai Chen,[a,1] Yonglun Zeng,[b,1] Hiu Yan Wong,[a,1] Yihong Chen,[a] Lei Yang,[a] Fang Luo,[c] Caiji Gao,[d] Liwen Jiang,[b*] Kam-Bo Wong[a*]

[a] Centre for Protein Science and Crystallography, [b] Centre for Cell and Developmental Biology, State Key Laboratory of Agrobiotechnology, School of Life Sciences, The Chinese University of Hong Kong, Shatin, Hong Kong, China

[c] Department of Developmental Biology, School of Basic Medical Sciences, Southern Medical University, Guangzhou, Guangdong 510515, China

[d]Guangdong Provincial Key Laboratory of Biotechnology for Plant Development, School of Life Sciences, South China Normal University, Guangzhou 510631, China

[1] These authors contributed equally to this article.

* To whom correspondence should be addressed.

Email:

Liwen Jiang ljiang@cuhk.edu.hk;

Kam-Bo Wong: kbwong@cuhk.edu.hk

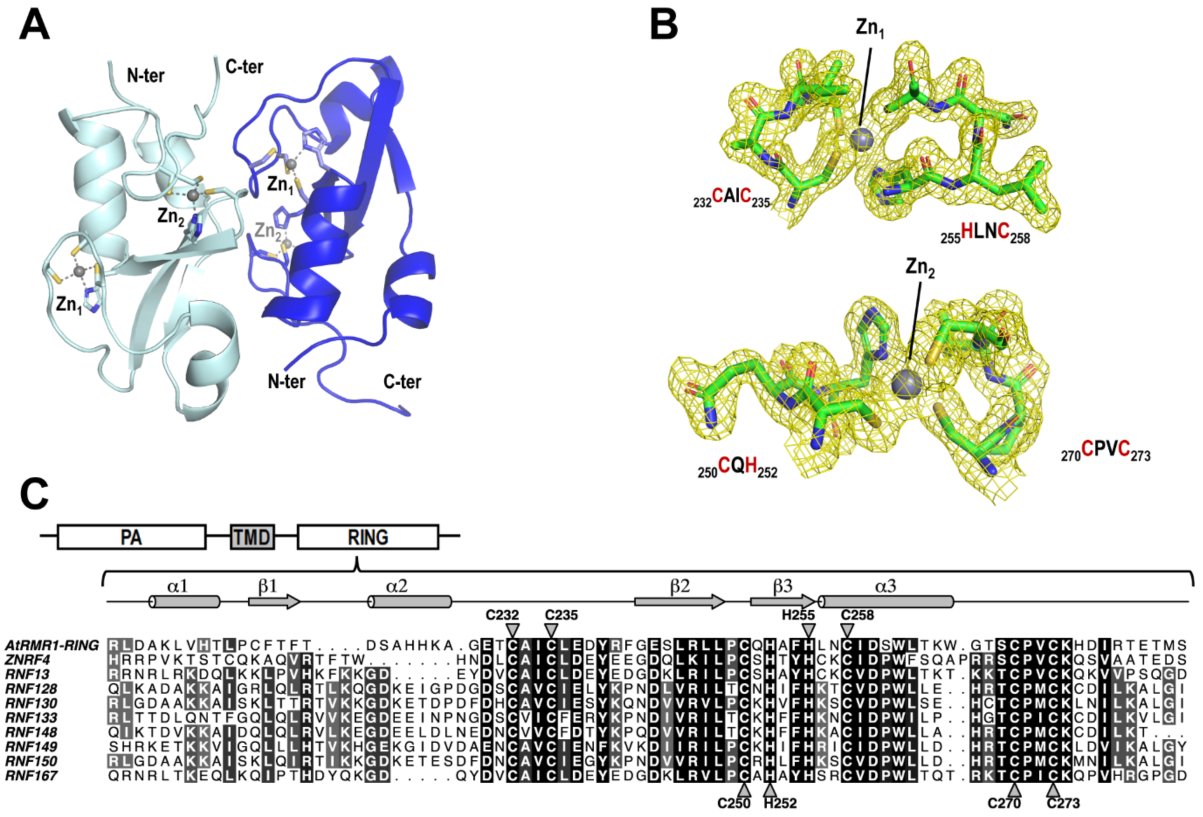

Figure S1. Coordination of two zinc ions by the C3H2C3 motif in AtRMR1-RING. (A) The crystallographic asymmetric unit contains two protomers (cyan and blue) of AtRMR1-RING, each binding two zinc ions. (B) Sigma-weighted 2Fo-Fc electron density map (contoured at 1σ) highlighting the two zinc-binding sites in AtRMR1-RING. Zn1 is coordinated by Cys232, Cys235, His255, and Cys258, while Zn2 is coordinated by Cys250, His252, Cys270, and Cys273. These residues form the C3H2C3 motif. (C) The C3H2C3 motif is conserved in other proteins with the PA-TMD-RING domain organization. RMR contains a protease-associated domain at its N-terminal region and a RING-H2 domain at its C-terminal region. The sequence of AtRMR1-RING is aligned with the RING-H2 domain of other known E3 ligases (ZNRF4, RNF13, RNF128 RNF130, RNF133, RNF148, RNF149, RNF150, RNF167) with this PA-TMD-RING topology. Residues involved in binding Zn1 are indicated by inverted triangles and those in binding Zn2 are indicated by triangles. Residues are numbered according to the sequence of AtRMR1. The secondary structure elements of the resolved AtRMR1-RING structure are shown above the sequence alignment.

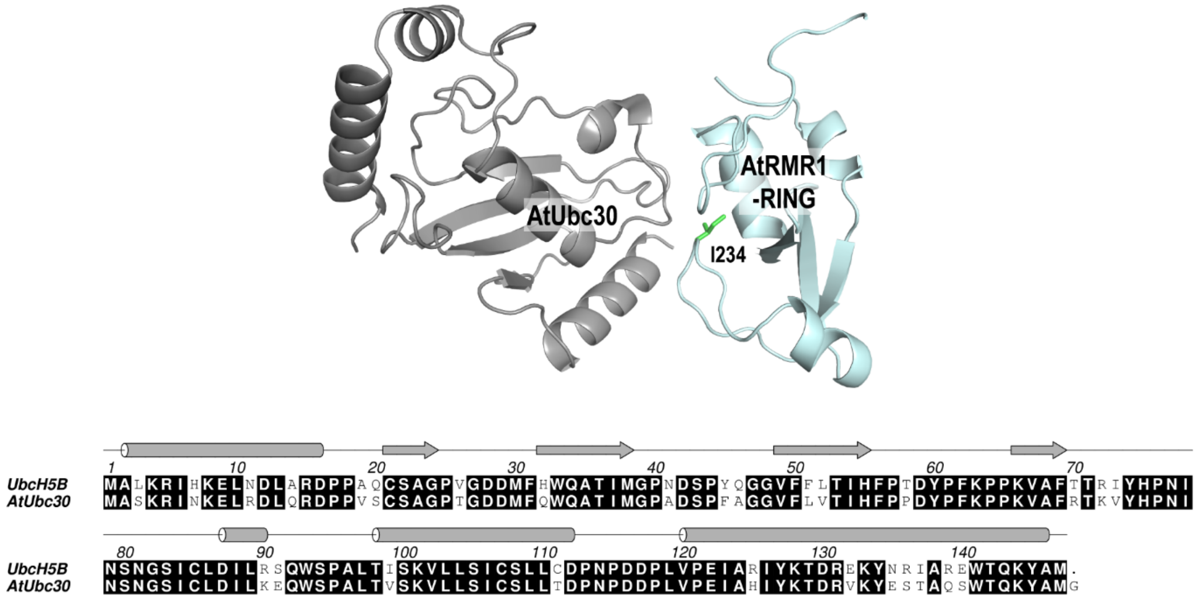

Figure S2. Modelling of the complex structure of AtRMR1-RING/AtUbc30. The structure of AtUbc30 is predicted using homology modelling with the crystal structure of human UbcH5B as a template. The crystal structure of AtRMR1-RING and the predicted structure of AtUbc30 are juxtaposed according to the crystal structure of RNF38-UbcH5B-Ubiqutin complex (PDB code: 4V3K). As shown in the sequence alignment, AtUbc30 shares 79% sequence identity to UbcH5B.

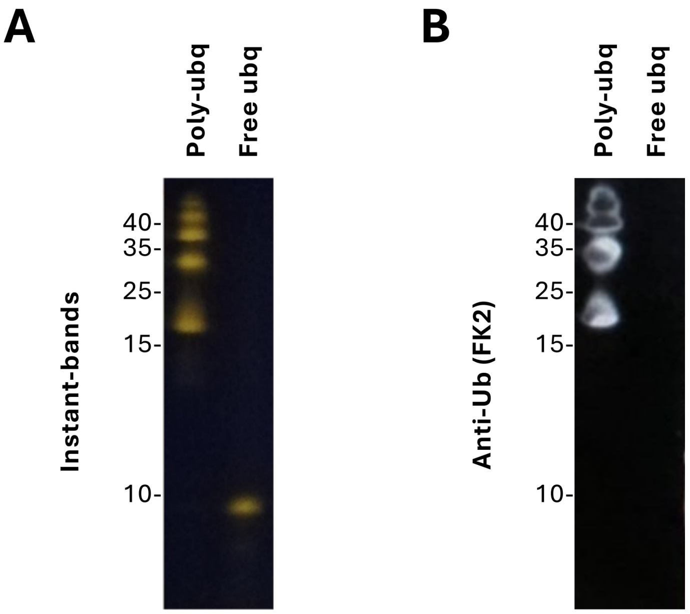

Figure S3. The FK2 ubiquitin antibody detects poly-ubiquitin but not free ubiquitin. Poly-ubiquitin and free ubiquitin were resolved on a 16% Tricine-SDS-PAGE. They were detected by (A) Instant-Bands (EZBiolab) that stains proteins fluorescently and (B) Western blot using the FK2 ubiquitin antibody (ENZO Biochem). We showed that the FK2 antibody detected only the poly-ubiquitin but not the free ubiquitin.

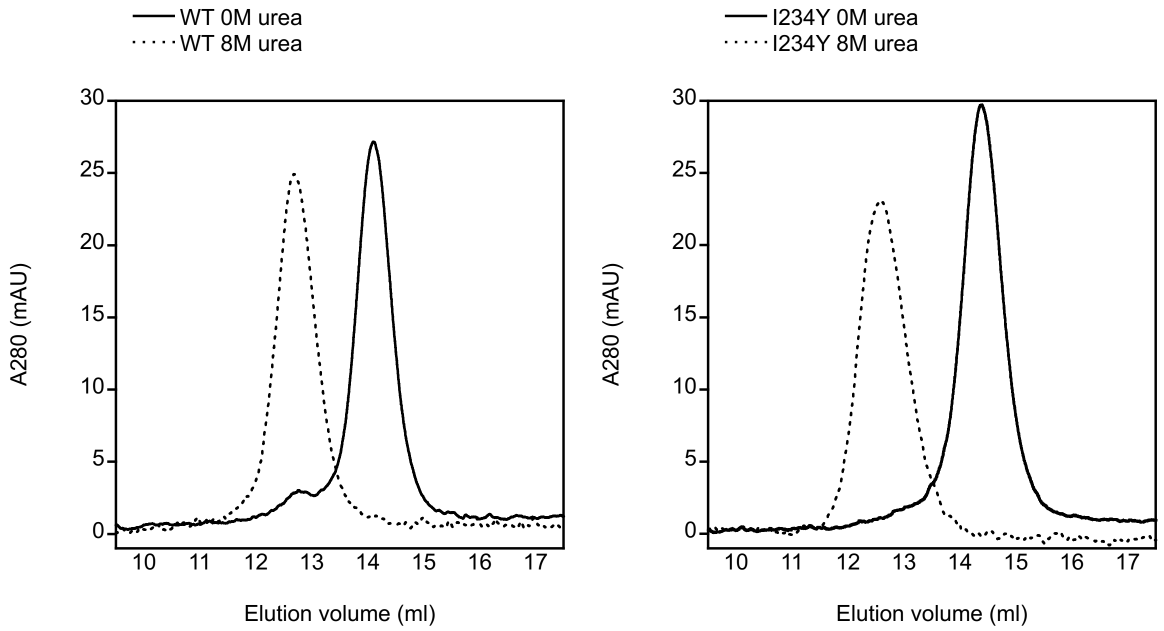

Figure S4. Gel filtration analysis showed that the I234Y variant of AtRMR1-RING is folded. 100 µL 0.3 mg/ml wild-type (A) or I234Y variant (B) of AtRMR1-RING were loaded to a Superdex 75 Increase 10/300 GL column (GE Healthcare) pre-equilibrated with 0M or 8M urea in a running buffer (30 mM Tris, pH 7.5, 150 mM NaCl and 2 mM DTT). The elution volume for I234Y variant was 14.4 ml, which is similar to that for WT, suggesting the I234Y variant was folded. When 8M urea was added to the protein samples and the running buffer, the elution volumes of WT and I234Y variant (dotted lines) were shifted to 12.7 and 12.6 ml, respectively.

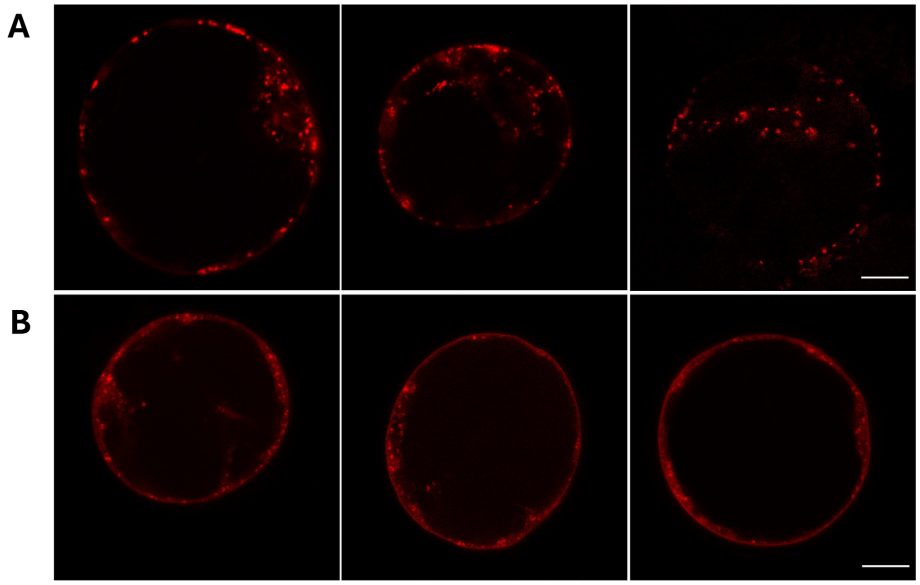

Figure S5. Expression of the Goligi and TGN markers in PSBD cells. *Arabidopsis* protoplasts were transfected with the Golgi (ManI-mRFP) (A) or TGN (mRFP-SYP61) (B) markers before confocal imaging of transfected cells. Scale bar =10 μm.

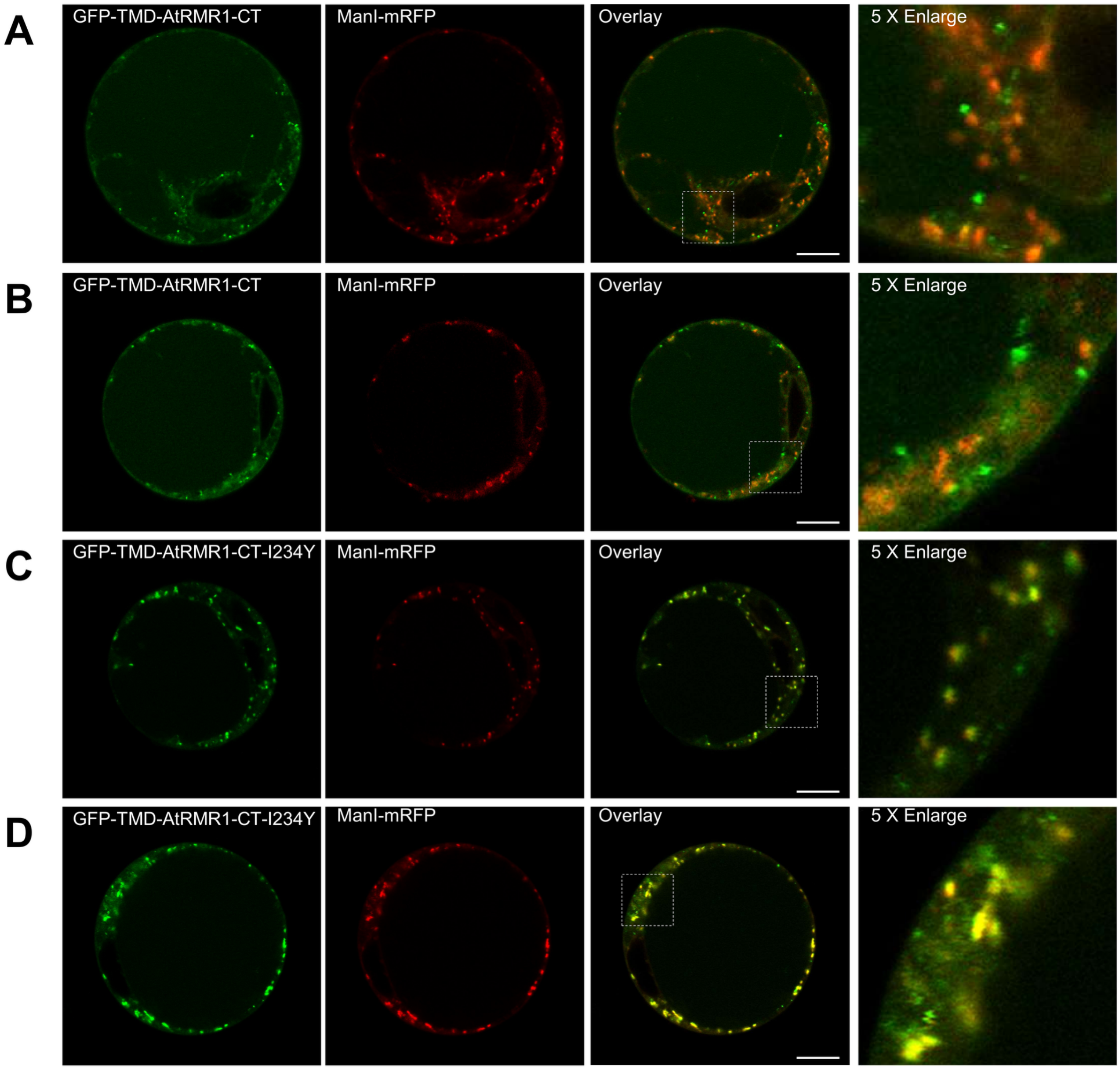

Figure S6. Confocal images from additional biological repeats used in the colocalization analysis of GFP-TMD-AtRMR1-CT and the Golgi marker. *Arabidopsis* protoplasts were transfected with (A and B) wild-type or (C and D) I234Y mutant of GFP-TMD-AtRMR1-CT and the Golgi marker (ManI-mRFP) before confocal imaging of transfected cells. (Scale bar, 10 μm).

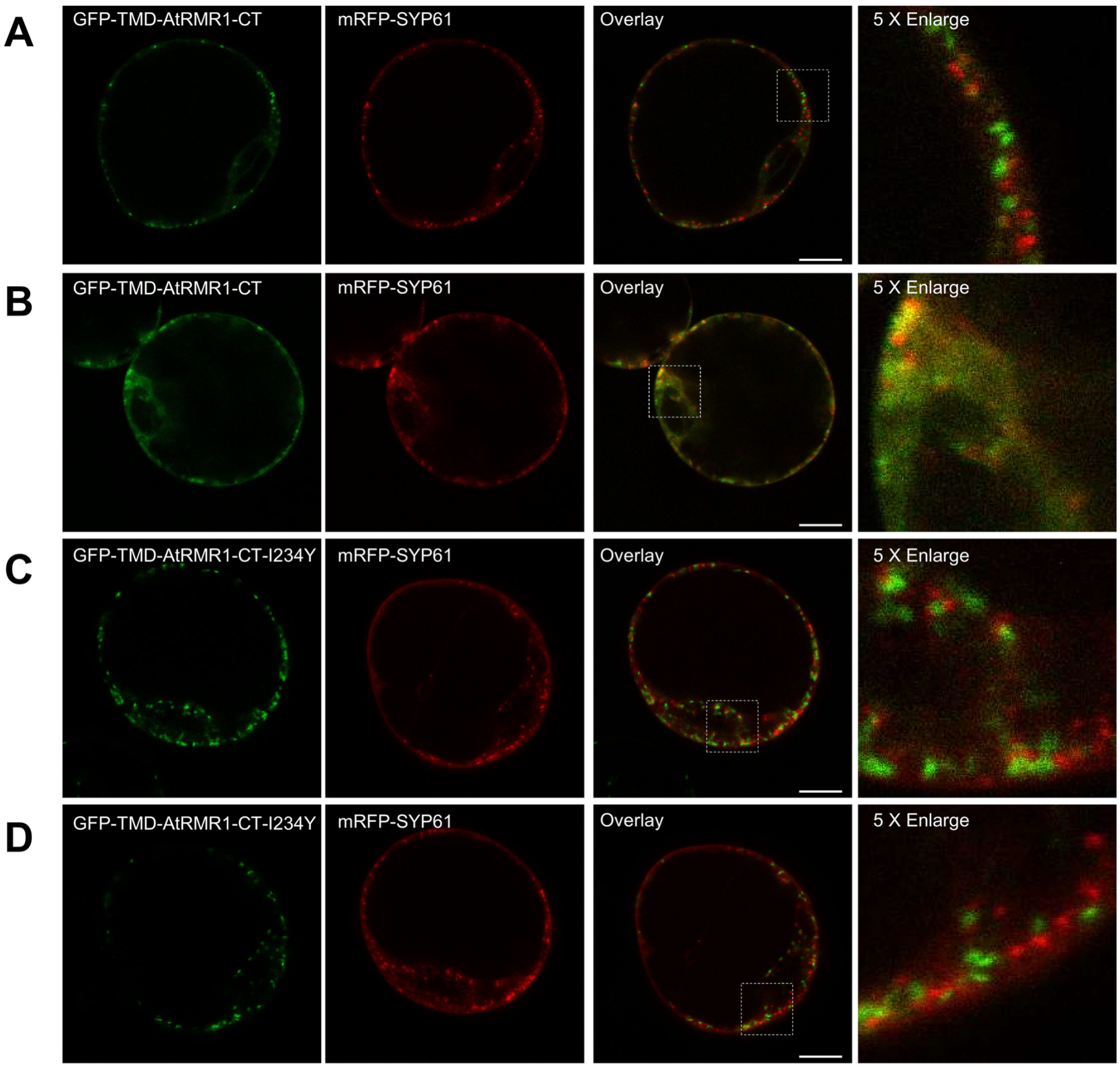

Figure S7. Confocal images from additional biological repeats used in the colocalization analysis of GFP-TMD-AtRMR1-CT and the TGN marker. *Arabidopsis* protoplasts were transfected with (A and B) wild-type or (C and D) I234Y mutant of GFP-TMD-AtRMR1-CT and the TGN marker (mRFP-SYP61) before confocal imaging of transfected cells. (Scale bar, 10 μm).

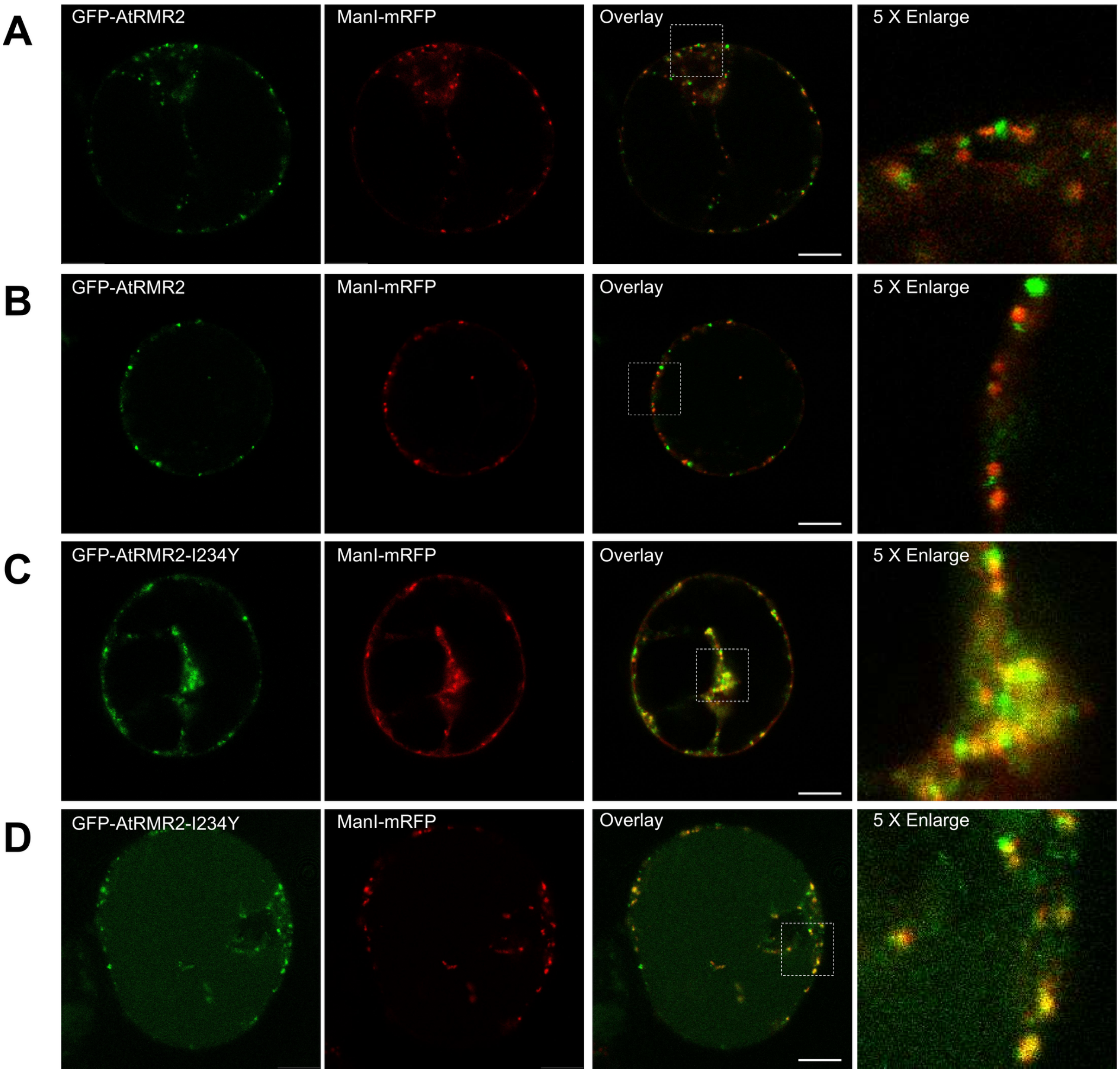

Figure S8. Confocal images from additional biological repeats used in the colocalization analysis of GFP-AtRMR2 and the Golgi marker. *Arabidopsis* protoplasts were transfected with (A and B) wild-type or (C and D) I234Y mutant of GFP-AtRMR2 with the Golgi marker (ManI-mRFP) before confocal imaging of transfected cells. (Scale bar, 10 μm).

A

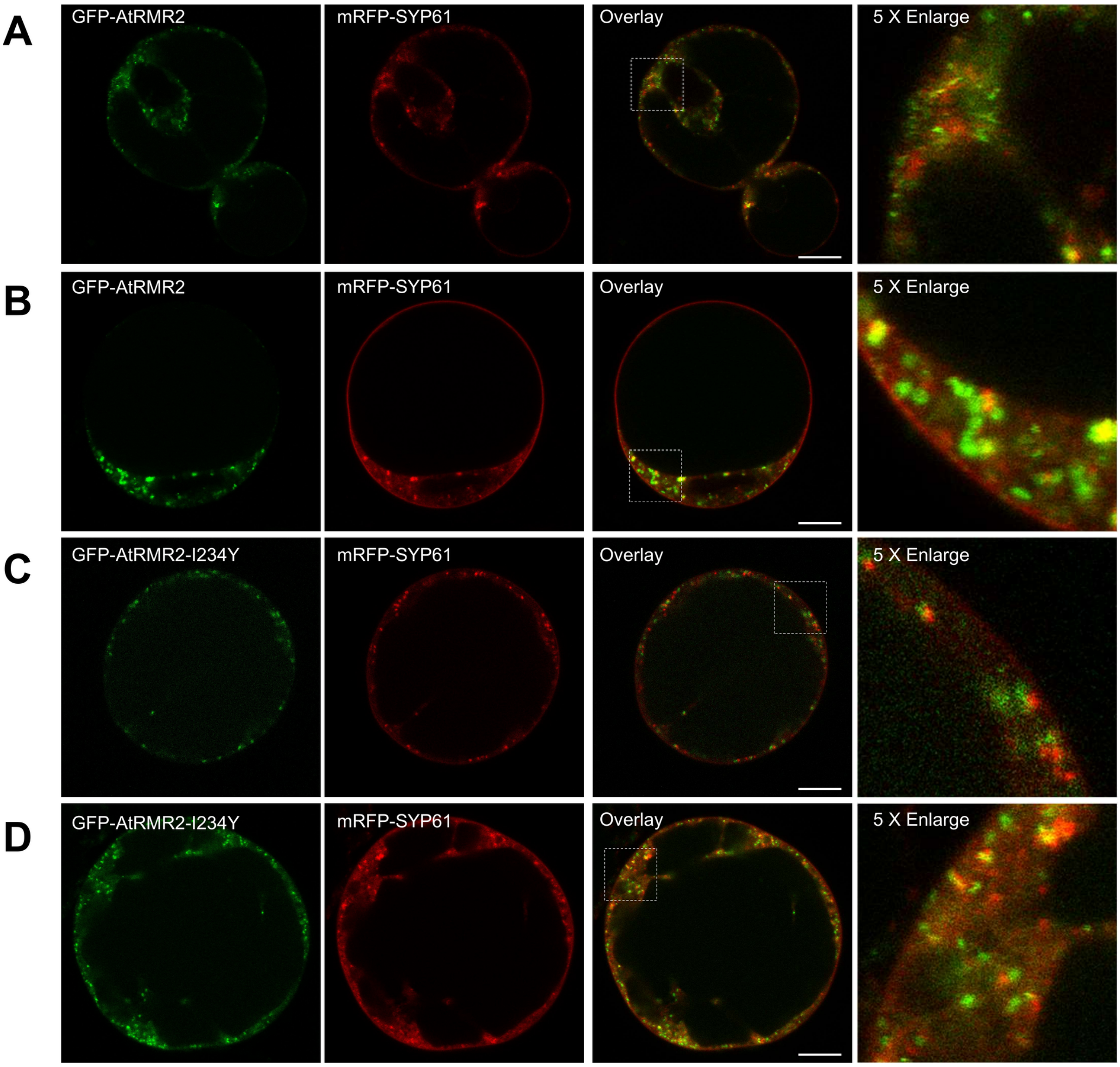

Figure S9. Confocal images from additional biological repeats used in the colocalization analysis of GFP-AtRMR2 and the TGN marker. *Arabidopsis* protoplasts were transfected with (A and B) wild-type or (C and D) I234Y mutant of GFP-AtRMR2 with the TGN marker (mRFP-SYP61) before confocal imaging of transfected cells. (Scale bar, 10 μm).

Table S1. Data collection and refinement statistics of AtRMR1-RING

**Data Collection**	
Space group	C2
Cell dimensions	
a, b, c (Å)	74.431, 23.648, 75.699
α, β, γ (°)	90, 94.8, 90
Resolution (Å)	37.72-2.10 (2.16-2.10)
No. of reflections	7849 (650)
R_{merge} (%)	8.5 (19.1)
I/σ (I)	9.0 (5.0)
Completeness	98.5 (97.4)
Multiplicity	3.6 (3.4)
**Refinement**	
R_{work}/R_{free} (%)	17.91/22.19
No. of atoms	1,263
Protein	1,184
Water	73
Metal	6
r.m.s. deviation	
Bond lengths (Å)	0.007
Bond angles (°)	0.478
Clashscore	1.8
Ramachandran Plot	
Favored	98%
Allowed	2%
Outliers	0%

Table S2. Protein and Nucleotide sequences used in this study

Constructs	Protein sequence	Nucleotide sequence
AtUba1	EIDEDLHSRQLAVYGRETM	GAGATTGACGAAGATCTGCACAGCCGTCAACTGGCGGTTTACGGT
(72-1080)	RRLFASNVLISGMHGLGAE	CGTGAAACCATGCGTCGTCTGTTCGCGAGCAACGTGCTGATCAGC
	IAKNLILAGVKSVTLHDERV	GGCATGCATGGTCTGGGCGCGGAAATCGCGAAAAACCTGATTCTG
	VELWDLSSNFVFSEDDVG	GCGGGTGTGAAGAGCGTTACCCTGCACGACGAGCGTGTGGTTGA
	KNRADASVQKLQDLNNAV	ACTGTGGGATCTGAGCAGCAACTTCGTGTTTAGCGAGGACGATGT
	VVSSLTKSLNKEDLSGFQV	TGGCAAAAACCGTGCGGACGCGAGCGTTCAGAAGCTGCAAGATCT
	VVFSDISMERAIEFDDYCH	GAACAACGCGGTGGTTGTGAGCAGCCTGACCAAAAGCCTGAACAA
	SHQPPIAFVKADVRGLFGS	GGAAGACCTGAGCGGTTTCCAGGTTGTGGTTTTTAGCGATATCAG
	VFCDFGPEFAVLDVDGEE	CATGGAGCGTGCGATTGAGTTCGACGATTACTGCCACAGCCACCA
	PHTGIIASISNENQAFISCV	ACCGCCGATCGCGTTCGTGAAGGCGGACGTTCGTGGTCTGTTTGG
	DDERLEFEDGDLVVFSEVE	CAGCGTGTTCTGCGATTTTGGTCCGGAGTTCGCGGTGCTGGACGT
	GMTELNDGKPRKIKSTRPY	TGATGGCGAGGAACCGCACACCGGCATCATTGCGAGCATCAGCAA
	SFTLDEDTTNYGTYVKGGI	CGAGAACCAGGCGTTTATTAGCTGCGTTGACGATGAACGTCTGGA
	VTQVKQPKLLNFKPLREAL	GTTCGAAGACGGTGATCTGGTGGTTTTTAGCGAGGTGGAGGGTAT
	KDPGDFLFSDFSKFDRPPL	GACCGAGCTGAACGACGGCAAACCGCGTAAGATCAAAAGCACCCG
	LHLAFQALDHFKAEAGRFP	TCCGTATAGCTTCACCCTGGACGAAGATACCACCAACTACGGCAC
	VAGSEEDAQKLISIATAINT	CTATGTTAAGGGTGGCATTGTGACCCAGGTTAAACAACCGAAGCT
	GQGDLKVENVDQKLLRHF	GCTGAACTTTAAGCCGCTGCGTGAGGCGCTGAAGGACCCGGGTG
	SFGAKAVLNPMAAMFGGIV	ATTTCCTGTTTAGCGACTTCAGCAAATTTGATCGTCCGCCGCTGCT
	GQEVVKACSGKFHPLFQF	GCACCTGGCGTTCCAGGCGCTGGACCACTTTAAAGCGGAAGCGG
	FYFDSVESLPSEPVDSSDF	GTCGTTTCCCGGTGGCGGGCAGCGAGGAAGATGCGCAAAAGCTG
	APRNSRYDAQISVFGAKFQ	ATCAGCATTGCGACCGCGATCAACACCGGTCAGGGCGACCTGAAA
	KKLEDAKVFTVGSGALGCE	GTGGAGAACGTTGATCAAAAGCTGCTGCGTCACTTCAGCTTTGGC
	FLKNLALMGVSCGSQGKL	GCGAAGGCGGTTCTGAACCCGATGGCGGCGATGTTTGGTGGCATT
	TVTDDDIIEKSNLSRQFLFR	GTGGGTCAGGAAGTGGTTAAAGCGTGCAGCGGCAAGTTCCACCC
	DWNIGQAKSTVAASAAAVI	GCTGTTTCAATTCTTTTACTTCGACAGCGTTGAGAGCCTGCCGAGC
	NPRFNIEALQNRVGAETEN	GAACCGGTGGACAGCAGCGATTTTGCGCCGCGTAACAGCCGTTAT
	VFDDAFWENLTVVVNALD	GACGCGCAGATCAGCGTTTTCGGTGCGAAATTTCAAAAGAAACTG
	NVNARLYVDSRCLYFQKPL	GAGGATGCGAAGGTGTTCACCGTTGGTAGCGGCGCGCTGGGTTG
	LESGTLGTKCNTQSVIPHL	CGAATTTCTGAAAAACCTGGCGCTGATGGGCGTTAGCTGCGGTAG
	TENYGASRDPPEKQAPMC	CCAGGGCAAACTGACCGTGACCGACGATGACATCATTGAGAAGAG
	TVHSFPHNIDHCLTWARSE	CAACCTGAGCCGTCAGTTCCTGTTTCGTGACTGGAACATCGGCCA
	FEGLLEKTPAEVNAYLSSP	AGCGAAGAGCACCGTGGCGGCGAGCGCGGCGGCGGTTATCAACC
	VEYTNSMMSAGDAQARDT	CGCGTTTCAACATTGAGGCGCTGCAAAACCGTGTGGGTGCGGAAA
	LERIVECLEKEKCETFQDC	CCGAAAACGTTTTCGATGACGCGTTTTGGGAAAACCTGACCGTGG
	LTWARLRFEDYFVNRVKQ	TTGTGAACGCGCTGGACAACGTGAACGCGCGTCTGTACGTTGATA
	LIYTFPEDAATSTGAPFWS	GCCGTTGCCTGTATTTTCAGAAACCGCTGCTGGAAAGCGGCACCC
	APKRFPRPLQYSSSDPSLL	TGGGCACCAAGTGCAACACCCAAAGCGTTATCCCGCACCTGACCG
	NFITATAILRAETFGIPIPEW	AGAACTACGGTGCGAGCCGTGACCCGCCGGAAAAACAGGCGCCG
	TKNPKEAAEAVDRVIVPDF	ATGTGCACCGTGCACAGCTTCCCGCACAACATTGATCACTGCCTG
	EPRQDAKIVTDEKATTLTT	ACCTGGGCGCGTAGCGAGTTTGAAGGCCTGCTGGAGAAGACCCC
	ASVDDAAVIDDLIAKIDQCR	GGCGGAAGTTAACGCGTACCTGAGCAGCCCGGTGGAGTATACCAA
	HNLSPDFRMKPIQFEKDDD	CAGCATGATGAGCGCGGGTGATGCGCAGGCGCGTGATACCCTGG
	TNYHMDVIAGLANMRARN	AGCGTATCGTGGAATGCCTGGAGAAGGAAAAATGCGAAACCTTTC
	YSIPEVDKLKAKFIAGRIIPAI	AAGATTGTTTAACCTGGGCGCGTCTGCGTTTCGAAGATTACTTTGT
	ATSTAMATGLVCLELYKVL	GAACCGTGTTAAACAACTGATTTATACCTTTCCGGAAGATGCGGCG
	DGGHKVEAYRNTFANLAL	ACCAGCACCGGTGCGCCGTTCTGGAGCGCGCCGAAGCGTTTTCC

	PLFSMAEPLPPKVVKHRD<br>MAWTVWDRWVLKGNPTL<br>REVLQWLEDKGLSAYSISC<br>GSCLLFNSMFTRHKERMD<br>KKVVDLARDVAKVELPPYR<br>NHLDVVVACEDEDDNDVDI<br>PLVSIYFR	GCGTCCGCTGCAGTACAGCAGCAGCGACCCGAGCCTGCTGAACTT<br>CATCACCGCGACCGCGATTCTGCGTGCGGAAACCTTTGGTATCCC<br>GATTCCGGAATGGACCAAAAACCCGAAAGAGGCGGCGGAAGCGG<br>TTGACCGTGTGATCGTTCCGGATTTCGAGCCGCGTCAAGACGCGA<br>AAATTGTGACCGATGAAAAAGCGACCACCCTGACCACCGCGAGCG<br>TGGATGATGCGGCGGTTATCGATGACCTGATCGCGAAAATTGACC<br>AGTGCCGTCACAACCTGAGCCCGGATTTCCGTATGAAACCGATTC<br>AATTTGAGAAGGATGACGATACCAACTACCACATGGACGTTATCGC<br>GGGTCTGGCGAACATGCGTGCGCGTAACTATAGCATTCCGGAAGT<br>GGATAAGCTGAAAGCGAAGTTCATCGCGGGTCGTATCATTCCGGC<br>GATTGCGACCAGCACCGCGATGGCGACCGGCCTGGTGTGCCTGG<br>AGCTGTACAAAGTTCTGGACGGTGGCCACAAGGTGGAAGCGTATC<br>GTAACACCTTCGCGAACCTGGCGCTGCCGCTGTTTAGCATGGCGG<br>AGCCGCTGCCGCCGAAAGTTGTGAAGCACCGTGACATGGCGTGG<br>ACCGTGTGGGATCGTTGGGTTCTGAAAGGTAACCCGACCCTGCGT<br>GAGGTTCTGCAGTGGCTGGAAGACAAGGGTCTGAGCGCGTACAG<br>CATCAGCTGCGGCAGCTGCCTGCTGTTCAACAGCATGTTTACCCG<br>TCACAAAGAGCGTATGGATAAGAAAGTTGTGGACCTGGCGCGTGA<br>TGTGGCGAAGGTTGAACTGCCGCCGTACCGTAACCACCTGGACGT<br>TGTGGTTGCGTGCGAGGATGAAGACGATAACGACGTGGATATCCC<br>GCTGGTTAGCATTTATTTCCGT
AtUbc30<br>(1-148)	MASKRINKELRDLQRDPPV<br>SCSAGPTGDDMFQWQATI<br>MGPADSPFAGGVFLVTIHF<br>PPDYPFKPPKVAFRTKVYH<br>PNINSNGSICLDILKEQWSP<br>ALTVSKVLLSICSLLTDPNP<br>DDPLVPEIAHIYKTDRVKYE<br>STAQSWTQKYAMG	ATGGCGAGCAAGCGTATCAACAAAGAGCTGCGTGACCTGCAGCGT<br>GATCCGCCGGTTAGCTGCAGCGCGGGTCCGACCGGCGACGATAT<br>GTTCCAGTGGCAAGCGACCATCATGGGTCCGGCGGACAGCCCGT<br>TTGCGGGTGGCGTGTTTCTGGTTACCATTCACTTCCCGCCGGATTA<br>CCCGTTTAAGCCGCCGAAAGTGGCGTTTCGTACCAAGGTTTATCAC<br>CCGAACATCAACAGCAACGGCAGCATCTGCCTGGACATTCTGAAG<br>GAACAATGGAGCCCGGCGCTGACCGTGAGCAAAGTTCTGCTGAGC<br>ATTTGCAGCCTGCTGACCGACCCGAACCCGGATGATCCGCTGGTG<br>CCGGAGATCGCGCACATTTACAAGACCGATCGTGTTAAATATGAAA<br>GCACCGCGCAGAGCTGGACCCAAAAATACGCGATGGGT
AtUb<br>(1-76)	MQIFVKTLTGKTITLEVESS<br>DTIDNVKAKIQDKEGIPPDQ<br>QRLIFAGKQLEDGRTLADY<br>NIQKESTLHLVLRLRGG	ATGCAGATTTTTGTGAAGACCCTGACCGGCAAGACCATTACCCTGG<br>AAGTTGAAAGCAGCGACACCATTGATAACGTGAAAGCGAAAATCCA<br>GGACAAAGAGGGTATCCCGCCGGATCAGCAACGTCTGATCTTCGC<br>GGGTAAACAACTGGAAGACGGCCGTACCCTGGCGGATTACAACAT<br>TCAAAAGGAGAGCACCCTGCATCTGGTTCTGCGTCTGCGTGGCGG<br>T
AtRMR1-<br>RING (206-<br>283)	RLDAKLVHTLPCFTFTDSA<br>HHKAGETCAICLEDYRFGE<br>SLRLLPCQHAFHLNCIDSW<br>LTKWGTSCPVCKHDIRTET<br>MS	CGTCTGGACGCGAAGCTGGTGCACACCCTGCCGTGCTTCACCTTT<br>ACCGATAGCGCGCACCACAAAGCGGGTGAAACCTGCGCGATTTGC<br>CTGGAAGACTACCGTTTTGGTGAAAGCCTGCGTCTGCTGCCGTGC<br>CAACACGCGTTTCACCTGAACTGCATCGACAGCTGGCTGACCAAG<br>TGGGGTACCAGCTGCCCGGTGTGCAAACACGATATTCGTACCGAAA<br>CCATGAGC
AtRMR2-<br>RING (207-<br>283)	SRRLVKAMPSLIFSSFHED<br>NTTAFTCAICLEDYTVGDKL<br>RLLPCCHKFHAACVDSWLT<br>SWRTFCPVCKRDARTSTG<br>E	AGCCGTCGTCTGGTTAAGGCGATGCCGAGCCTGATCTTCAGCAGC<br>TTTCACGAGGACAACACCACCGCGTTTACCTGCGCGATTTGCCTG<br>GAAGACTACACCGTTGGCGATAAGCTGCGTCTGCTGCCGTGCTGC<br>CACAAATTTCATGCGGCGTGCGTGGACAGCTGGCTGACCAGCTGG<br>CGTACCTTTTGCCCGGTTTGCAAACGTGATGCGCGTACCAGCACC<br>GGCGAG

AtRMR3-RING (207-282)	CRRTVKAMPSVTFTCAKIDNTTGFSCAICLEDYIVGDKLRVLPCSHKFHVACVDSWLISWRTFCPVCKRDARTTAD	TGCCGTCGTACCGTGAAGGCGATGCCGAGCGTTACCTTCACCTGCGCGAAAATTGACAACACCACCGGTTTTAGCTGCGCGATCTGCCTGGAGGACTATATTGTGGGCGATAAGCTGCGTGTTCTGCCGTGCAGCCACAAATTCCACGTGGCGTGCGTTGATAGCTGGCTGATCAGCTGGCGTACCTTTTGCCCGGTGTGCAAACGTGATGCGCGTACCACCGCGGAT
AtRMR4-RING (209-284)	PKSMIIRMPTTIFNGICDEATTSILCCICLENYEKGDKLRILPCHHKFHVACVDLWLGQRKSFCPVCKRDARSIST	CCGAAGAGCATGATCATTCGTATGCCGACCACCATCTTTAACGGCATTTGCGATGAGGCGACCACCAGCATCCTGTGCTGCATTTGCCTGGAGAACTACGAAAAGGGTGACAAACTGCGTATCCTGCCGTGCCACCACAAGTTCCACGTGGCGTGCGTTGATCTGTGGCTGGGCCAGCGTAAGAGCTTTTGCCCGGTGTGCAAACGTGACGCGCGTAGCATTAGCACC

Table S3. Constructs and primers used in this study

Constructs / Uses	Primer sequence (5' to 3')
**pGEX-6p-1-AtRMR1-RING**<br>Subcloning of AtRMR1-RING (206-283) into the vector pGEX-6p-1 for E. coli expression.	CGCGGATCCCGTCTGGACGCGAAGCTG
	ACGCGTCGACTCAGCTCATGGTTTCGGTACG
**pGEX-6p-1-AtUba1**<br>Subcloning of AtUba1 (72-1080) into the vector pGEX-6p-1 for E. coli expression.	CGCGGATCCGAGATTAGACGAAGATCTG
	ACGCGTCGACTCAACGGAAATAAATGCTAAC
**pGEX-6p-1-AtUbc30**<br>Subcloning of AtUbc30 into the vector pGEX-6p-1 for E. coli expression.	CGCGGATCCATGGCGAGCAAGCGTATC
	ACGCGTCGACTCAGCCCATCGCGTATT
**pGEX-6p-1-AtUb**<br>Subcloning of AtUb into the vector pGEX-6p-1 for E. coli expression.	CGCGGATCCATGCAGATTTTTGTG
	ACGCGTCGACTCAACCGCCACGCAG
**GFP-TMD-AtRMR1-CT**<br>Subcloning of TMD-AtRMR1-CT (166-310) into the vector pBI221-AluSP-GFP for transient expression in Arabidopsis protoplasts	CTAGTCTAGAGCTTGGACTGTGTTG
	CGAGCTCCTAACGGCTTTGACTG
**GFP-AtRMR2**<br>Subcloning of AtRMR2 (22-448) into the vector pBI221-AluSP-GFP for transient expression in Arabidopsis protoplasts	CTAGTCTAGAGTTATTTTGATGAGGAATAACATC
	CGAGCTCCTAACAGTCTGGAAGCGAG
**AtRMR1-RING-I234Y**<br>To introduce I234Y mutation in pGEX-6p-1-AtRMR1-RING	AACCTGCGCGTACTGCCTGGAAGAC
	TCACCCGCTTTGTGGTGC
**GFP-TMD-AtRMR1-CT-I234Y**<br>To introduce I234Y mutation in GFP-TM-AtRMR1-CT	AACATGTGCTTACTGTCTCGAGGATTACAGATTTGGAGAAAGCCTCAG
	TCCCCGGCCTTGTGGTGA
